# Lost in turbulence? Healthcare workers’ conceptualisations and experiences with navigating time in personalised care

**DOI:** 10.1108/JHOM-07-2024-0295

**Published:** 2025-02-14

**Authors:** Teresa Corbett, Ninna Meier, Jackie Bridges

**Affiliations:** School of Health Sciences, University of Southampton, Southampton, UK; NIHR ARC Wessex, Southampton, UK; Department of Sociology and Social Work, Aalborg Universitet, Aalborg, Denmark

**Keywords:** Personalised care, Healthcare professionals, Qualitative research, Healthcare system, Time, Temporal patterns, Standardisation, Workload, Older adults, Aged, Multimorbidity, Professional-patient relations

## Abstract

**Purpose:**

The study aims to explore how healthcare workers (HCWs) navigate and experience time when caring for older cancer patients living with other illnesses.

**Design/methodology/approach:**

This paper presents findings from a qualitative study of how HCWs conceptualise and navigate the temporal aspects of delivering personalised care to older people living with multimorbidity. Building on research from organisation studies and the sociology of time, we interviewed 19 UK HCWs about their experiences of delivering care to this patient group.

**Findings:**

Our findings illustrate how the delivery of personalised care contradicts contemporary models for healthcare delivery defined by efficiency and standardisation. We found that HCWs engage with time as both a valuable commodity to be rationed and prioritised within a constrained context and as a malleable resource for managing workload and overcoming “turbulence” in the system. However, participants in this study also shared how the simultaneous multiplicity and lack of time had a profoundly personal impact on them through the emotional toll associated with “time debt” and “lost” time.

**Originality/value:**

This research presents a unique analysis of how time is conceptualised and navigated in contemporary healthcare, offering valuable insights for policy improvement. We conclude that personalised models of healthcare are incompatible with many current temporal structures of treatment trajectories and work-practices, by nature of being centred around the person and not the system of delivery.

## Introduction

As managerial reach has broadened to include the management of healthcare, many health systems in high income countries have approached the design and delivery of care with a view to standardising practices (Bohmer, 2009; Pinder *et al*., 2006). These approaches draw on scientific management principles about how work standardisation leads to predictability, consistency in quality and the efficient distribution of resources (Pinder *et al*., 2006; Schein, 2017). They are based on assumptions of correctly characterised single health problems and certainty about the care that will lead to the best outcomes (Bohmer, 2009). These standardised practices are specified in guidelines, protocols and pathways, that, until relatively recently, were developed to address single health conditions. More broadly, the “when”, “how often”, “how long”, “in what order” and “at what speed” of healthcare activities are handled in standardized allocations of time for appointments, exams and procedures that are made visible and managed through a variety of technologies. “Clock time” dominates here with multiple standardised plans dictating what work should happen when and clinical performance evaluated against the pre-set time parameters. Clock time is decontextualised and measurable, and is perceived as universal and objective (Adam, 1995; Caldeira and Timmins, 2015; Clissett *et al*., 2013; Hendrich *et al*., 2008).

Healthcare is considerably more variable than these approaches would suggest, and in fact markedly more differentiated care is needed at an individual patient level in many cases (Berg, 1997, 1999; Bohmer, 2009). Old age and multiple morbidities are very likely to add complexity to the nature of healthcare need and uncertainty in the clinical encounter as to the care that will result in optimal health (Bridges *et al*., 2019). There are differences between individuals in disease types, symptom profiles and progression, in addition to differences in how these elements interact with each other and with personal characteristics such as age-related biological changes, values, health literacy and available resources including social networks (Bridges *et al*., 2019). When an individual has complex needs, effective care is personalised, that is specifically tailored to that individual in that moment (Bridges *et al*., 2019). Where healthcare is taking place in a context of constraint where, for instance, time, information and/or skills are more limited than the interaction demands, an iterative (rather than standardised) approach to care is required that enables personalised care that accommodates the contingencies of that particular context (Berg, 1997, 1999; Bohmer, 2009). Process time, rather than clock time, is highly relevant here. Process time is fluid and context dependent (Davies, 1994; Urry, 2000). The inherent flexibility enables the practitioner to continuously adapt to the needs of the older person and contexts within which they work (Nowotny, 1992). Such “necessary improvisation” allows healthcare practitioners to deviate from standardised practice where appropriate and is a hallmark of good quality care (Bourdieu, 1977).

Changes in the structure, governance and organisation of healthcare systems can be viewed temporally (Meier, 2019). Healthcare systems are necessarily pluritemporal, consisting of many temporal structures and paces, and they are inhabited by people – staff, patients and others whose relationships with time tend be biographical, embodied and highly individual (Adam, 1995). Research to date has helped uncover the tensions between the deployment of scientific management principles to guide how care is organised and the realities facing health care professionals as they encounter patients with very complex needs (for instance Berg, 1997, 1999; Meier, 2011; Colley *et al*., 2012). However, even though clock time is a conspicuous feature of scientific management principles and process time appears to be a necessary component of personalised care, contemporary studies that view these tensions and their impact through a temporal lens are less common.

Herein, we explore how healthcare workers (HCWs) conceptualise time and navigate multiple temporalities in the provision of care for older adults with complex health needs. Drawing on an exploratory study using qualitative methods, we show how HCWs engage with time as both a valuable commodity to be rationed and prioritised within a constrained context and as a malleable resource for managing workload and overcoming “turbulence” in the system. However, participants in this study also shared how the simultaneous multiplicity and lack of time had a profoundly personal impact on them through the emotional toll associated with “time debt” and “lost” time.

## Literature review: temporality, standardisation and care

### Temporalities in care work

Organisational structures and processes aimed at streamlining and optimising organisation of care and treatment are a common feature of contemporary healthcare. To support the division and coordination of work in healthcare organisations, work processes and patient pathways are ostensibly organised according to time. The tempo and pace of care, or the speed at which events take place, has increased significantly in recent years (Aiken *et al*., 2012; Willis *et al.*, 2015). Quicker has often been equated with better quality, and such understandings of quality are usually accompanied by significant focus on reducing waiting and treatment times (Kreindler, 2010).

Accelerated or “fast care” (Rushton *et al*., 2016) is perceived to be vital to reducing waiting times and meeting targets (Bevan and Hood, 2006). Fast care is associated with a high-pace and quick turnover, with HCWs having interactions with an increased number of patients over a reduced period, thus serving a larger group of people. However, accelerated care may have harmful impact on individuals, as those receiving care may perceive interactions as rushed potentially leading to a reluctance to express doubts, concerns and additional care needs (Bridges *et al*., 2020; Hope *et al*., 2022; Moyle *et al*., 2015). As healthcare work is fundamentally uncertain and people’s responses to treatment (as well as their needs and preferences) can vary significantly (Bohmer, 2009), HCWs must be able to be pragmatic, adaptive and responsive in their work. This can often be at odds with health care work systems that emphasise speed as a priority. In contrast to fast care, “slow care” refers to engagement that is maintained over a longer period, centred on longitudinal awareness of the patient’s need.

Different illnesses have distinct trajectories, which relate not only to the physiological unfolding of a patient’s disease, but also to the total organisation of work done over that course, plus the impact on those involved with that work and its organisation (Strauss *et al*., 1985). As such, much of healthcare does not easily align with abstract clock time or standardised, pre-defined allocation of resources and when only certain, quantifiable, aspects of healthcare work are measured, other aspects of work tend to be overlooked or become functionally invisible from a systems perspective (Meier, 2011). Yet, standardisation and optimisation processes can create unintended and undesirable organisational effects, especially where individuals have multiple co-occurring health conditions (Pederson and Obling, 2020).

### Standardisation

From a scientific management perspective efficiency in care should be achievable through standardisation (Schein, 2017). This standardisation has typically been sought through the introduction of multiple care pathways and protocols, designed to streamline treatment and care and optimise use of time. Although these tools aim to ensure equal access to care based on “best available evidence”, they work in several ways when implemented into clinical micro settings, often with unintended consequences, requiring substantial amounts of additional work (Meier, 2011). Administrative doctrines described as New Public Management (NPM; Hood, 1991, 1995) seek to achieve efficient and effective delivery of services, and value-for-money. NPM is centred on principles of disaggregation or separation of the purchaser and provider of services, incentivisation and competition or choice (Lindaas *et al*., 2024). However, NPM has been criticised for promoting “cost-cutting” rather than effectiveness (Watson, 2003). When healthcare is considered primarily through the lens of protocolised work, reducing the time and cost of providing care may be incentivised and prioritised over optimising the quality of the care provided (Bone, 2002; Colley *et al*., 2012). De Menezes (2023) highlights a risk of perceived unrealistic and/or ambiguous targets, evaluations unrelated to performance-objectives, unethical behaviours by employees striving to meet expectations and performance-measures that are meaningless. Glaser and Strauss (1968) noted long ago that in hospitals, prescribed schedules govern care, and there is much evidence to suggest that this remains the case in many settings (for instance, Bridges *et al*., 2020). This routinisation of care is illustrated in attempts to standardise practices through protocols, guidelines, audited practices and through a wide variety of technologies monitoring compliance. McCormack and McCance (2011) noted that healthcare systems place a greater emphasis on aspects that can be quantified (such as time taken), with few indicators that are personalised. Aspects of “soft skills”, relational care and emotional work are under-valued, despite potentially impacting quantifiable metrics by promoting good quality care.

Core values of the NPM, that is competition, choice and service differentiation can contradict goals of equity and universality of service provision (Fotaki, 2010). Simonet (2015) notes that NPM theories view recipients of public services as customers rather than passive recipients, noting that this approach may promote social exclusion. Complex cases where patients have requirements that extend beyond medical needs (e.g. social problems, language or literacy barriers and limited access to transport) can be left behind as they are not equipped to advocate or empowered to make the best use of medical information and alternatives presented to them (Simonet, 2015). Standardising methods such as protocols, by their nature, prescribe the best way to treat and care for a type of patient. They are general, not situated and thus cannot account for context, nor unexpected or practical eventualities which may exert greater urgency than the protocol. They are coordination mechanisms best-suited to relatively stable settings such as elective surgery or out-patient care in certain specialities, where our knowledge of the nature and course of illness is well-established (Bohmer, 2009). Protocols for managing health trajectories can be highly prescriptive (Berg, 1997, p. 1083). However, human bodies are complex, characterised by “temporal unpredictability” (Cohen, 2011) and can respond in a variety of ways to different interventions. Protocols frequently fail to capture the nuances and complexities of healthcare, such as the emotional work that clinicians engage in. Such work is more difficult to quantify using clock time, and in turn, is often overlooked, unaccounted for or even viewed as a luxury (Bridges *et al*., 2013).

Healthcare delivery, research and education was built, and mostly remains centred, on the treatment of single diseases (Kingston *et al*., 2018). Cohen (2011) argued that a focus on a single part of the body or single disease (termed “standardisation by selection”) can be a deliberate strategy for managing complexity and limiting variation in the bodily functions of concern. Health service provision for those with multiple conditions is often duplicative, fragmented, inefficient and ineffective because of poor coordination and integration (Barnett *et al*., 2012). The co-occurrence of multiple complex conditions increases the likelihood of hospital admission, length of stay and readmission, raises healthcare costs, reduces quality of life and increases dependency, polypharmacy and mortality (Kingston *et al*., 2018). Complete adherence to existing clinical practice guidelines is not realistic, leading to inevitable poor adherence, wasted resources and poor outcomes. Yet, a lingering acute-care mentality, derived from the previous era of the acute illness, is still deeply influential in modern healthcare. Indeed, some have indicated that personalised care has become more about maintaining the current, medically led system and changing the patient, than about changing the system to meet the needs of individuals (Byrne, 2022).

### Ambitions of personalised care

Recent guidelines for the management of multiple co-occurring conditions emphasise personalised care, characterised by proactive, interactive and inherently flexible approaches (Skou *et al*., 2022). Personalised care is associated with improved care experiences and outcomes, increased satisfaction among care providers and improved performance of health care organisations (Rushton *et al*., 2016). However, the approach appears to contradict clock time which privileges fast care, and numerous barriers to the implementation of personalised care have been identified. These include established practices and traditional work-structures, lack of confidence and perceived capability of the HCW, as well organisational factors that may disable rather than facilitate efforts to implement personalised care in practice (Moore *et al*., 2017). Systems that prioritise quantifiable tasks, schedules and procedures are inherently misaligned with modern policy recommendations that promote individualised, personalised and holistic approaches to care (Jones, 2001).

The implementation of personalised care is set against a background of crises in healthcare systems in relation to increased waiting times, treatment cancellation rates, iatrogenic injury and clinical workloads in many healthcare systems (Alderwick and Dixon, 2019). Rushton *et al*. (2016) note that “shortage of time” is often cited as reason why HCWs feel unable to provide personalised care, particularly in acute care settings. A perception of “time debt” (Waterworth, 2003; Menzies, 1960) can lead to adoption of strategies to manage the psychological stress associated with healthcare work (Menzies, 1960). One such strategy is a tendency to revert to highly routinized care to reconcile competing temporal demands. Strategies that aim to alleviate time debt can often prioritise the interests of the organization rather those of person receiving care, resulting in poorer outcomes (Clissett *et al*., 2013; Edvardsson *et al*., 2014; Nilsson *et al*., 2013).

### Multiple simultaneous temporalities in healthcare

As discussed previously, clock time is fixed, measured and predictable and therefore well-suited as a shared conceptuliasation of time by which activities may be organised and coordinated. In contrast, “process time” is time that is fluid and context dependent. In process time, beginnings and endings are flexible and undefined (Davies, 1994; Urry, 2000). Davies (1994) noted that process time is central to care provision. Process time is particularly compatible with the concepts of “slow care” and personalised care outlined above. Obedience to clock time at the expense of process time can constrain healthcare delivery; yet many healthcare tasks necessitate a recognition of clock-time. An approach to reconcile both is “pluritemporalism”, which facilitates flexibility between context, persons and situations. Pluritemporalism (Nowotny, 1992), refers to the idea that there can be multiple “times” simultaneously co-occurring. The concept of pluritemporalism is particularly relevant to discussions of personalised care, and the idiosyncratic nature of older people’s care.

Pederson and Obling (2020) note that time is often implicit in sociological studies of healthcare standardisation, and Egede-Nissen *et al*. (2013) call for research that explores the relationship between good quality care and time use. The study reported here addresses a particular gap in knowledge in this field, addressing the issues in practice arising from an increasingly aged and multimorbid population. This study focuses on the embodied experiences of HCWs addressing the temporal dimensions of care in contexts where old age and multimorbidity feature. HCW accounts of their experiences are used to illustrate how HCWs conceptualise time and navigate multiple temporalities in these contexts.

## Design and methods

The paper draws on exploratory qualitative one-to-one interview data collected as part of a larger study aiming to develop an intervention to promote personalised care and support self-management in older adults with complex conditions (Corbett *et al*., 2022). The focus of this analysis is HCWs’ experiences of time when providing care to older people with multiple health conditions alongside a diagnosis of cancer.

### Data collection

We conducted one-to-one qualitative interviews with 19 HCWs from the National Health Service in England between March and August 2019. Interviewees worked in a variety of roles for a range of providers in primary and secondary healthcare (See Table 1). Face-to-face interviews were carried out at a convenient location and lasted around 90 min. All interviews were audio-recorded and fully transcribed. A semi-structured interview guide was developed collaboratively with HCWs and a patient representative. Although we based our interview guide on theories of workload and capacity (Shippee *et al*., 2012; May *et al.*, 2009), we adjusted questions iteratively throughout the interview process, carefully considering the experience and context of the interviewees. The research project was approved by the University of Southampton ethics committee.

**Table 1:** Overview of interviewees

Ppt ID	Gender	Role
107	Female	Advanced Clinical Practitioner in Frailty
102	Female	Allied Health Professional
120	Female	Lead Cancer Nurse
115	Female	Cancer Support Worker
117	Female	Cancer Support Worker
119	Female	Cancer Support Worker
116	Female	Cancer Nurse Specialist
110	Male	Consultant Haematologist
103	Male	Consultant in Geriatric Medicine
112	Female	Consultant in Pain
105	Female	Consultant Nurse for Frailty
111	Male	Frailty and Older Persons Rapid Assessment Unit
108	Female	Cancer Support Worker
109	Female	Long Term Conditions Lead Nurse
104	Female	Cancer Nurse Specialist
113	Female	Nurse Consultant for Older Person’s Mental Health
118	Male	Oncology Nurse
114	Female	Research Nurse
106	Female	Specialist Respiratory Nurse

**Source(s):** Authors’ work

### Data analysis process

TC independently read the transcripts and identified recurring ideas and concepts. The data were analysed using systematic reading, familiarisation and open coding. Initial ideas were discussed by all three authors to explore initial themes and refine the connections. Following this, we drew on literature on standardisation and temporality to help us to make sense of the findings. This provided a backdrop of concepts that help to sensitise us to possibilities in our data. We focused our analysis on how the participants in this study conceptualised time and navigated tensions in relation to temporality to facilitate the practice of good quality care.

In line with Rushton *et al*.’s (2016) advice against dichotomous representations of time, we recognised that a dualistic focus could limit ones understanding by emphasising the tensions between different temporal structures. Therefore, we began by discussing the results of the initial thematic analysis in relation to a variety of conceptualisations of time to analyse in depth how HCWs navigate the multiple temporal aspects of delivering care. These included clock time, sovereign time, pluritemporalism and process time. We used the concepts of fast care and slow care (Aiken *et al*., 2012; Nilsson *et al*., 2013) and discussed how these conceptualisations of care related to the different engagements with time that we identified in our material. We also considered the implications of a healthcare system mostly defined by clock time and characterised by invariance, context independence and precision (Rushton *et al*., 2016). We drew on the work of Elias (1992, 2000) to establish an experiential understanding of time and temporal synchronisation challenges. This enabled us to explore concepts such as coordination and synchronisation processes within and between different settings. We also studied the work of Berg (1997, 1999) and Bohmer and Lee (2009) who discussed scenarios whereby protocols can dictate timings which do not align with the realities of healthcare interactions. We explored the idea of “managing patients’ trajectories” as a central aspect of healthcare work practices. The concept of turbulence (Jenning *et al.*, 2013, 2022) was likewise influential in our understanding of the data, helping us to consider the consequences of misalignment within the system. These considerations led us to compare temporal tensions in how work-is-imagined (or promoted by policy) and how work-is-done, drawing on work by Braithwaite *et al*. (2017). Further, we considered the tension between the temporal ordering assumed as feasible in a work system that is task-based and the fluid, thoroughly contextual nature of “body time” (e.g. Cohen, 2011; Twigg, 2006). We adjusted the coding considering these sensitising concepts identified in the literature. Themes were developed from the codes, and iteratively compared and modified through discussion amongst the co-authors. This led us to question our initial assumptions about the skillsets or capabilities to engage in personalised care practices, to focus on how healthcare providers reconcile the fluid and flexible nature of personalised care within time-poor healthcare systems characterised by standardised pathways and single-disease focused approaches to care.

## Findings

While healthcare systems remain organised largely in line with clock-time, our findings suggests that HCWs engage with temporality in several nuanced ways. We found that HCWs conceptualise and navigate time as both a valuable commodity to be rationed and prioritised within a constrained context and as a malleable resource for managing workload and overcoming “turbulence” in the system. However, participants in this study also shared how the simultaneous temporal multiplicity and lack of time had a profoundly personal impact on them through the emotional toll associated with “time debt” and “lost” time, as we unfold below.

### Time debt: time is a valuable finite resource that can be used, but also overspent

**Figure 1:**
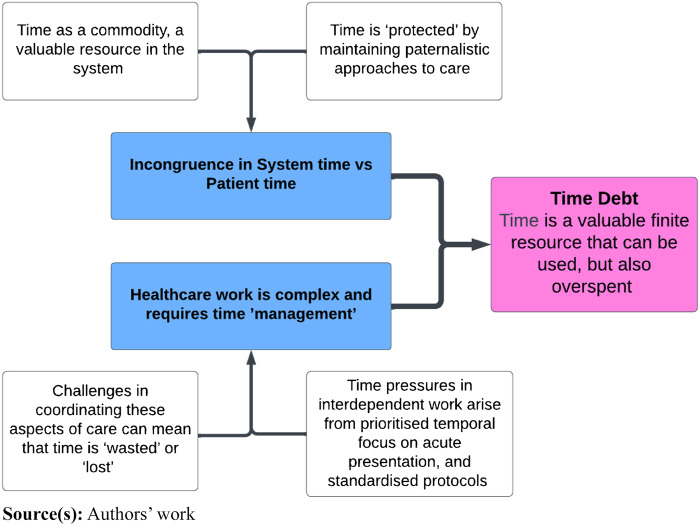
Time debt: time is a valuable finite resource that can be used, but also overspent

Time was conceptualised as a commodity by the individuals we interviewed, a valuable resource of the organisation that necessitated prioritisation and rationing (Figure 1).

Conflicts over time control were associated with the duration of the working day, the pace of work and break times, overtime and time off. For many participants, there was a perceived need to engage in continual boundary work to manage their own (and patients’) time, behaviours and emotions. These experiences demonstrated a pressurised context of constraint where time, information and knowledge about the presenting case is more limited than the interaction demands:

I think, we’re all at saturation point and there are only so many new assessments and new tools and new recording systems that one can do before actually you spend your entire life nursing paper and not people …. There are just never enough hours in the day/week and, you know, you just have to work out what you don’t do and invariably that will be, you know, the paperwork and the stuff that’s not directly patient facing … - PPT 116

Cohen (2011) argues that standardisation is desirable in healthcare because it facilitates the predictable allocation of resources. With little time to spare, power is retained by HCWs maintaining paternalistic approaches to care and avoiding scenarios that may take more time. However, the deliberate deconstruction of the person into “manageable” units is often incongruent with models of person-centred care and multimorbidity. Following standardised protocols assumes high certainty as to the right course of action. HCWs in this study illustrated how the work of managing multimorbidity in older age requires working with a high level of uncertainty, and of experimenting with what may or may not be the right course of action.

Findings reflect that working with older people living with multimorbidity was complex and labour intensive. Challenges in coordinating aspects of care associated with multiple health conditions meant that time felt wasted or lost, both for the practitioner and the individual receiving care. Participants provided examples of how protocol-driven over-treatment led to escalation that could reduce the older adults’ quality of life rather than improve it. They cited examples of how this could result in prolonged hospital stays and unnecessary invasive procedures, both of which may be costly to a healthcare system where time resources are scarce. Health-related work was described as being pushed back to the older person and their family to reconcile the misalignment in the system, adding to the emotional impact and cognitive burden of living with complex needs:

If I get older, I’d want to get older without lots of conditions because however positive and robust old people are the accumulation of those deficits is a burden. And the burden of treating--- because we treat each individual things separately. It all becomes very muddled.--- I wouldn’t be able to cope with their medication regimes and I would not be able to cope with the number of people that talk to me. And yet, I expect a ninety-five year old with short term memory problems to do deal with it. -PPT 107

HCWs pointed out that co-occurring conditions were often only actively considered when something went wrong, rather than being addressed proactively. The temporal focus on the immediate was perceived as short-sighted, and often an inefficient use of resources. Participants recounted examples of how the immediate single diseases (sometimes perceived as fixable) took priority over the complexities of multimorbidity. Many highlighted a “*tendency to focus on the immediate problem*”. In turn, clinicians described their role as “*firefighting*.” Acute concerns were prioritised over the chronic, with little emphasis possible on forward planning to prevent or pre-empt future crises. HCWs also described how they needed to work in their own time to complete administration tasks that they had previously abandoned to address the acute needs of patients during their shift. Some indicated that they “*chose*” to engage in such work beyond their shift but noted that they rarely received recognition or reparation for doing so.

### Time is malleable (structuring/stretching/moving around)

**Figure 2:**
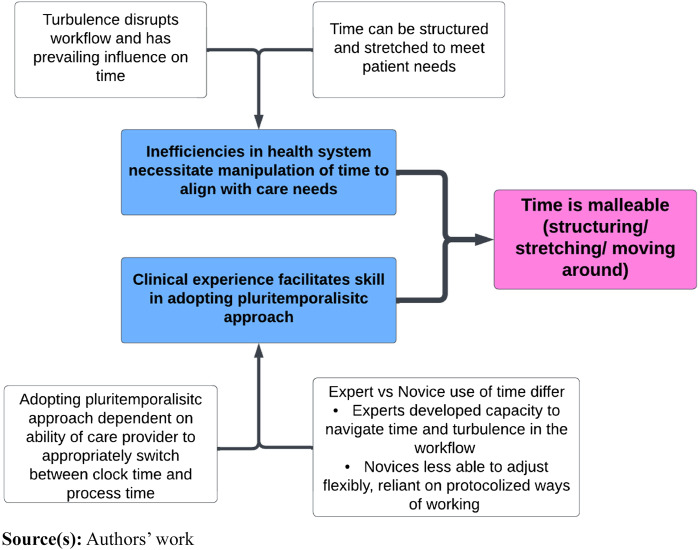
Time is malleable (structuring/stretching/moving around)

Participants also described time as malleable, providing examples of how time could be structured and stretched to align with care needs and clinician experience, see Figure 2 below.

Time pressure necessitated continuous management of numerous, often competing demands within the temporal boundaries of each shift. Constraints on time led to task switching (moving to a second task before finishing the first) and multitasking (doing two things at once; Walter *et al*., 2014). Reprioritising (Patterson *et al.*, 2011) was used to avoid waiting when delays were created by system delays. Cognitive stacking (Sitterding *et al*., 2012) was employed as a strategy for managing workload when multiple demands occurred simultaneously. In addition to these tactics, participants also adopted protocolised and boundaried approaches to care to “manage” interactions with the person receiving care. Perceived constraints of time (and other resources) meant that there was often a tendency to keep the interaction limited to what is manageable in the available time.

Those with established experience of healthcare work with this client group described how they had developed a capacity to reconstruct trajectories to provide person-centered care, manage turbulence in the workflow, while also leading to more efficient use of their time. This reflected a pluritemporalistic approach, balancing the demands of clock time while optimising personalised care. More experienced clinicians described themselves as proactive and less reliant on protocolised ways of working. These individuals described how they had developed an ability to responsively alter their plans to ensure patient safety, as well as flexibility in approaching or delaying certain tasks.

Conversely, for less experienced or junior colleagues, working in a pluritemporalistic way was viewed as more challenging. They were reported as being less able to adjust their practices to the changing requirements of situations. These individuals were depicted as focusing efforts on basic task-oriented clinical skills, perhaps indicating a lack of confidence to adopt more ad hoc, personalised approaches where necessary. This aligns with Clissett *et al*. (2013) observation of occasions when health professionals needed to spend more time with an individual but felt unable to because of a perceived lack of time. Our findings suggest that effective “switching” between clock time and process time may be difficult for novice HCWs because they have not yet learned to navigate competing temporal structures (clock time versus process time, slow care versus fast care).

Structure is something that [novice HCWs] find really useful. And forms they find very useful because they’re used to being in that ward environment where, you know, literally almost now you don’t think because your dashboard tells you what to do. You don’t use intuition because you don’t need to because there is another form further in this packet which will tell you what to do. So, that knowledge and experience, I think, for a lot of younger nurses now they almost don’t see it as valuable because there’s always a form to make them safe and if the form’s filled in, it’s all alright. - PPT 116

Preparatory work was viewed as a key element in providing good quality, personalised care. While gathering this information takes time at the outset, it was generally seen (by experienced practitioners) as saving time later. However, participants frequently explained how much of this preparatory and administration work was “invisible” or unaccounted for. This time-consuming work was further exacerbated by inefficiencies in the healthcare system. For example, care was impacted by inconsistent reporting, incomplete records and cases where certain reviews did not happen routinely or were administered in a seemingly ad hoc or case-by-case basis. Consequently, participants described this process as a form of “detective work”, alluding to the challenging and often tedious nature of the task.

It is about being a detective because there are so many conditions this can be potentially--- so many medications … it’s about getting a really good history … and understanding of their background, of their conditions. Trawling through their notes.. You know, that you can really have a good look … I hate going in cold ….I think, staff will always see it as a very time consuming--- I see it as frontloading. I see it as: “get this right in the first place you save, you know, a lot of time later”. You get a better relationship with your patient early on. But, I think, for staff out at the coalface they’d have difficult--- difficulty crafting that care plan and they will always say they haven’t got time to.- PPT 105

Information about an individual with multiple conditions needed to be sought from various sources, some of which may be inaccessible due to disjointed systems within and between organisations. HCWs observed the variability in response and timeliness from colleagues asked to provide information, with some services being significantly less responsive. Online systems were described as difficult to use and as an inefficient way to share information. Distinct platforms and a range of different care records made it difficult to find information. Inaccessibility of information made it difficult for HCWs to ascertain the status of an individual receiving care, and in turn, to prepare for the consultation. They described having to meet individuals “blind”, not cognisant of other patient information, care plans and interventions. This lack of information threatened patient safety and could also lead to patient frustration or upset. Delays in receiving information also led to longer appointments, and a number noted that delays wasted time that could be spent identifying solutions for the person receiving care.

High demand on services and shortcomings in the work system created a turbulent environment in which to work. This turbulence disrupted and complicated workflow in unnecessary ways, and HCWs found their workload increased, pressuring the amount of time available. Interruptions in communication, inadequate handoffs, information overload and impaired decision-making due to a lack of information impeded HCWs’ capacity to practice personalised care. Turbulence was further boosted by large patient caseloads, high patient turnover, simultaneous demands, time pressures and issues with equipment and supplies, as well as inadequate space to hold personalised conversations.

### Time is personal and felt

**Figure 3:**
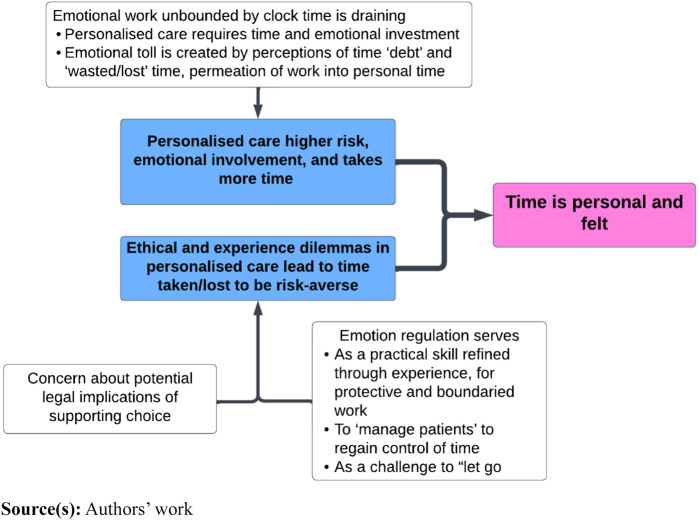
Time is personal and felt

In our third theme, we discuss the feelings of time, the emotional toll created by perceptions of time debt and “lost” time, see Figure 3 below.

When the valuable resource of time was “wasted”, work permeated into personal time. HCWs reflected on the challenge of “switching off” at the scheduled shift end-time, indicating that clinician shifts were not necessarily bounded by clock time. Healthcare work is therefore not only restricted to clinical tasks; it also permeates complex psycho-social phenomena such as emotional and cognitive work. Consequently, turbulence within the system may increase healthcare professional’s stress, burnout and diminish well-being (Jennings, 2021).

Emotion regulation was considered a practical skill that is refined through experience, perhaps facilitated through a process of “ongoing intersubjective surveillance and self-regulation” (Douglas-Creed *et al*., 2014, p. 275). Setting clear boundaries on the remit of their role and adopting structured approaches to care were viewed as protecting the clinician. Clinicians spoke of how they “manage patients” to regain control of time, sometimes deviating from personalised practices to address turbulence in the workflow. For example, one mentioned that if you asked someone how they were managing their health *“you might not get out of the room*”, perhaps illustrating the internalising of the institutional requirement that time is managed efficiently.

Participants noted how they find themselves continually mentally engaging with work, even after their shift-has ended. Several participants noted that it was a challenge to “*let go*” and help people take ownership of their healthcare. Some described feeling over-involved and described taking actions beyond the remit of their role to support older adults. Those who worked in specialised care settings raised concerns about overstepping, or being unsure about whether it was appropriate for them to intervene on issues that may be explored by another practitioner.

We would be on the ‘phone to each other all the time: “but..” or: “I’ve done this but I’m not really sure”, “what if this patient deteriorates?”, “do you think I’ve made the right decision?”, “what would you have done differently?” … you’ll always go home with (…) at the end of the day with certain patients and you’re not quite sure whether you’ve done the right or wrong thing. And that risk taking sometimes seems too much of a risk or too little of a risk. – PPT 111

Concern about potential legal implications left clinicians unsure about the extent to which they could truly support an individual’s choice, particularly if it did not align with expert opinion. Many raised concerns about challenges that could arise where the person’s priorities were incongruent with the clinician’s recommendations. Practitioners emphasised that it was important to strike the right balance between safeguarding and personal choice. Within a risk-averse and time-pressured healthcare system, documented medical assessments were viewed as central to care while the older person’s wishes were necessarily peripheral. Some participants viewed extensive record taking was a bureaucratic task in case legal issues arose, rather than as being for the benefit of service-users. They described administrative work as “*covering their back, ensuring you’ve a record*”. Fears of litigation and concerns about the time to engage in administrative tasks meant that clinicians were hesitant to transfer ownership to the person receiving care. However, participants who advocated personalised approaches noted that being risk-avoidant could lead to over-medicalisation and deviations from the person’s values and priorities. This was summarised by a participant who noted that what the older adult wants “*is lost*.” Such approaches to care ultimately disempower the person in receipt of care, impacting their time alongside their experience of care.

We often don’t do a particularly good job of finding out what’s important --- to the patients. And we often spend a lot of time imposing what’s important to us onto them. Sometimes, it’s appropriate and apparent and--- you know, it’s what you need to be doing. Sometimes it’s not. Other things can become lost in the pursuit of safety which is a shame. It’s not nice because you want to be doing what’s important to the person but you can become lost in the priorities of healthcare often won’t meet the priorities of the person and the priorities of the patient can become lost.- PPT 118

The HCWs we interviewed reflected that meaningful engagement with an individual’s specific priorities and needs entailed higher risk, emotional involvement and took more time. For many (especially less experienced practitioners), it was significantly less burdensome to opt for more prescriptive forms of care. Pre-established protocols and scripts closed conversations, saving time by reducing the need to engage in complex conversations and shared decision-making. Practitioners described feeling under pressure, noting that working in healthcare takes its toll emotionally, especially in relation to difficult and complex caseloads.

## Discussion

In this paper we have investigated how HCWs conceptualise and navigate time in the provision of care for older people with multi-morbidity. We have examined their views on the provision of “good” personalised care, as well as identifying aspects of care organisation that could impact how time is experienced in the healthcare system. Our analysis demonstrates how HCWs navigated the turbulence and temporal tensions of working with older adults living with multimorbidity by adopting a pluritemporalistic approach to achieve personalisation in care. Our temporal framing enabled us to theorise two main aspects of how tensions in the healthcare system are experienced by staff caring for people with complex health needs.

Firstly, inspired by Pedersen’s and Obling’s (2020) point that time is often implicit in studies of standardisation within medical sociology, we foregrounded temporal aspects of practicing care and demonstrate challenges associated with the prioritisation of accelerated and protocolised work processes. Specifically, we demonstrated an inherent misalignment between standardisation processes in healthcare systems and the fluidity required to engaged in personalised practices. Our study suggests that bureaucratic workloads and firefighting require time and attention away from care provision, while fragmented systems of communication absorb valuable time and resources. Participants in this study were generally positive towards the potential of personalised approaches to care, however, as found in previous research (e.g. Skou *et al*., 2022), many indicated implementation would require more time, skill and resource. These issues posed predominant barriers and undermined the implementation of personalised approaches for those with multiple long-term conditions.

Secondly, our foregrounding of temporality also allowed us to explore the emotional toll of balancing and attempting to accommodate these tensions within healthcare. Here, our findings demonstrate how protocolised working and boundaries can support healthcare workers, particularly those with less training and experience and that, in some instances, standardised practices supported a sense of order and safety. However, our results also suggest that highly personalised and flexible approaches may lead to perceptions of uncertainty, risk and anxiety, without the scaffolding of a prescribed approach. Participants frequently raised concerns about risk-taking, role boundaries and ethical concerns which demonstrate the “greyness” that comes with the inherent flexibility of personalised care provision. Without protocolised “black-and-white”, “if-then” solutions, clinicians are left to navigate complex cases independently in high-stress environments. In critiques of current policy and education relating to personalised care (Entwistle *et al.*, 2022), it has been reported that current training and policy models often fail to explicitly address the uncertainties and difficulties that HCWs may experience. There is a need to recognise and challenge tensions and practical-ethical issues that can arise in provision of personalised care (Entwistle *et al.*, 2022). Our findings indicate that a particular area for focus by policy makers is how to incentivise and adequately support the experimental risk-taking that may be required by HCWs to ascertain the best outcome for individuals with complex health needs.

With this paper, we build on literature that has sought to challenge the rhetoric of personalised care, by highlighting how healthcare systems inhibit personalised care practices. Byrne *et al*. (2024) note that tensions exist between HCWs' ability to deliver personalised care in a system designed to maintain NPM efficiency, productivity and efficacy. Instead, resource management is prioritised above safe, effective care (Ljungblom, 2014, p. 193). Consequently, care is ineffective and fragmented, characterised by processes that are overwhelming to both HCWs and patients. While personalised care is promoted to bridge some of these gaps (Byrne *et al*., 2024), it remains a challenge to implement in light of competing priorities. Cole *et al*. (2023) argue that the current regulatory ethos of healthcare is not supportive of the ideals of personalised care. Delivery of care is hampered by multiple layers of legislation, bureaucracy and standardisation. Stresses on efficiency, calculability, predictability and control create constraints associated with information overload, work intensification and role-conflict (McCann *et al.*, 2015). Austerity measures lead to staff reduction (Kasper *et al*., 2023), further intensifying the impact on HCW time. Instability and fragmentation can lead to increased incidences of missed care (Willis *et al*., 2017). Universally, the primary reasons for missed care are inadequate staffing levels, resource deficits and poor communication (Verrall *et al*., 2015), all of which we have discussed in the current paper. We have highlighted intentional decisions to ration, reprioritise or omit care as a result of “*multiple demands and inadequate resources*” (Kalisch *et al*., 2009, p. 1509). Consequently, although not explicitly mentioned, we can assume that the tensions we have outlined are likely to be associated with missed, reprioritised or delayed care. Patient care is compromised when routines are changed; when equipment is unavailable, and when staff are forced to seek it from another ward, or improvise (Henderson *et al.*, 2013). Lack of time is intensified by time spent managing turbulence, staffing levels that do not account for patient complexity and pressures that encourage HCWs to undertake tasks outside of their scope of practice and/or level of experience (Verrall *et al*., 2015). In turn, the intensification of labour and missed care affects employee wellbeing, patients' satisfaction and outcomes and operational performance (De Menezes, 2023; Schubert *et al*., 2009).

Personalised care can thus only be embedded within a system that is appropriately staffed, equipped and responsive. Workload models need to account for the nature of the modern patient, who is likely to present with multiple and chronic conditions. Capacity challenges due to inadequate staffing could be addressed by mandating minimum staff-patient ratios. These have been introduced in some contexts, leading to lower mortality rates (e.g. Aiken *et al*., 2010). Mentoring and clinical supervision could enable junior HCWs to develop personalised care skills in a supportive environment. Musker and Othman (2024) note that effective clinical supervision is associated with reductions in emotional exhaustion, depersonalisation and burnout. However, such initiatives require proactive investment in the workforce. Other initiatives could focus on prioritising care-work appropriately and reviewing the capabilities of those providing care (Verrall *et al*., 2015). There is also potential to rethink where personalised care interventions are implemented, shifting towards a more community-based model of care (Baird *et al*., 2024). A shift to primary and community services can reduce demand on hospitals, and improve integration of services (Baird *et al*., 2024).

### Strengths and limitations

This research was conducted between March and August 2019, and the participants relate to experiences prior to the onset of the Covid-19 pandemic. Our findings illustrate the vulnerability of the healthcare system prior to the pandemic, pointing to/highlighting substantial systemic barriers that have likely become further entrenched. Our interviews provided valuable insights into the experiences of those providing care as the NHS national long term plan was introduced. The experiences recounted here demonstrate that implementation of the plan would have always been ambitious. Given the timing of the research, and the country context, caution should be taken in transferring the results to other contexts.

This research is strengthened by the inclusion of a range of the healthcare providers, who shared experiences of the interdisciplinary nature of care for older people with complex conditions. Despite differences in roles and care settings, views shared across primary and secondary care complemented each other, strengthening the validity of our findings. The extensive use of theory provides a clearly articulated lens for how we approached the analysis, facilitating coherence and depth to enhance the explanatory power and legitimacy of our findings (Collins *et al*., 2018).

By analysing healthcare work using temporality theories, our work adds to the body of knowledge about how healthcare workers conceptualise time and how workers are able (or not) to manage tensions between the demands of multiple temporalities. We have also demonstrated the emotional toll on staff that this navigation and balancing requires, and how more standardised forms of care can reduce this toll by appearing to save time and reduce risk thus dis-incentivising the provision of personalised care. Our findings indicate that as populations age and health need become more complex, healthcare systems designed to support HCWs with the time, skill and resources to work effectively with uncertainty may enable the radical shift required from standardised ways of working to more responsive forms of care.
